# SPICY: a method for single scan rotating frame relaxometry†

**DOI:** 10.1039/d2cp05988f

**Published:** 2023-04-13

**Authors:** Katja Tolkkinen, Sarah E. Mailhiot, Anne Selent, Otto Mankinen, Henning Henschel, Miika T. Nieminen, Matti Hanni, Anu M. Kantola, Timo Liimatainen, Ville-Veikko Telkki

**Affiliations:** a NMR Research Unit, University of Oulu Oulu Finland ville-veikko.telkki@oulu.fi; b Department of Medicinal Chemistry, Uppsala University Uppsala Sweden; c Research Unit of Health Sciences and Technology, University of Oulu Oulu Finland; d Department of Diagnostic Radiology, Oulu University Hospital Oulu Finland; e Medical Research Center Oulu, University of Oulu and Oulu University Hospital Oulu Finland

## Abstract

*T*_1*ρ*_ is an NMR relaxation mode that is sensitive to low frequency molecular motions, making it an especially valuable tool in biomolecular research. Here, we introduce a new method, SPICY, for measuring *T*_1*ρ*_ relaxation times. In contrast to conventional *T*_1*ρ*_ experiments, in which the sequence is repeated many times to determine the *T*_1*ρ*_ time, the SPICY sequence allows determination of *T*_1*ρ*_ within a single scan, shortening the experiment time remarkably. We demonstrate the method using ^1^H *T*_1*ρ*_ relaxation dispersion experiments. Additionally, we combine the sequence with spatial encoding to produce 1D images in a single scan. We show that *T*_1*ρ*_ relaxation times obtained using the single scan approach are in good agreement with those obtained using the traditional experiments.

## Abstract 2

The SPICY NMR method enables the determination of *T*_1*ρ*_ relaxation times and 1D *T*_1*ρ*_ imaging in a single scan to elucidate low frequency molecular motions.

## Graphical abstract

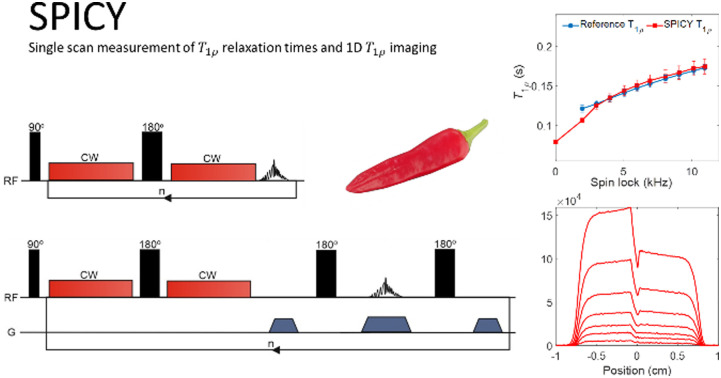

## 1. Introduction

Nuclear magnetic resonance (NMR) experiments provide versatile information on time-dependent dynamic processes of molecules. NMR relaxation experiments give information on molecular rotational motions and environments. Most often, NMR relaxation studies utilize longitudinal *T*_1_ and transverse *T*_2_ relaxation. *T*_1_ is sensitive to molecular motions with frequencies close to the Larmor frequency, *ω*_0_ = *γB*_0_, which is determined using gyromagnetic ratio *γ* and the strength of the static magnetic field *B*_0_.^1^ To probe a range of molecular motion frequencies, *B*_0_ needs to be changed,^2^ which is not possible with conventional NMR instruments.

However, there exists a third relaxation process called *T*_1*ρ*_, or spin–lattice relaxation in the rotating frame, where the nuclear magnetization returns to equilibrium under the influence of continuous wave spin lock (SL) field *B*_1_.^1,3,4^ Since the *ω*_1_ frequency can be changed, typically between 1 and 6 kHz, *T*_1*ρ*_ can be utilized to probe motional processes over a wide dynamic timescale.^4^ The *T*_1*ρ*_ relaxation time extends between *T*_2_ and *T*_1_ as the SL field increases, and this increase of *T*_1*ρ*_ is called *T*_1*ρ*_ relaxation dispersion.^4–7^

The other common method for measuring relaxation dispersion is *T*_2_ dispersion in which the effective transverse relaxation time is measured as a function of the time between the refocusing pulses in the Carr–Purcell–Meiboom–Gill (CPMG) pulse sequence.^8,9^ However, in conventional NMR instruments, *T*_2_ relaxation dispersion measurements are limited to lower frequency ranges than *T*_1*ρ*_ dispersion.^4,10^

Relaxation dispersion measurements have been shown to be a powerful tool for characterizing micro- and millisecond timescale biomolecular motions, such as chemical exchange and protein dynamics^4–7,11–16^ and biomechanical properties of objects like rat tissues^17,18^ and articular cartilage of various origins.^19–22^ Articular cartilage, which is a connective tissue possessing very specialized biomechanical properties, is composed of cartilage cells which are surrounded by an extracellular matrix, principally consisting of collagen, proteoglycans and water.^23^*T*_1*ρ*_ relaxation and dispersion studies have been utilized in both NMR spectroscopy and MRI to track biochemical changes of the cartilage extracellular matrix.^19–22,24–27^ An additional use of *T*_1*ρ*_ measurements is *T*_1*ρ*_-weighted magnetic resonance imaging (MRI).^28^*T*_1*ρ*_ preparation is often paired with MRI to obtain *T*_1*ρ*_ image contrast which provides higher resolution to some pathological changes and disease progression than only with *T*_1_ and *T*_2_ weighted imaging. For example, *T*_1*ρ*_ is sensitive to proteoglycan loss in articular cartilage as osteoarthritis progresses and *T*_1*ρ*_ mapping can detect early articular cartilage degradation with more sensitivity than *T*_2_ mapping.^29–32^ Furthermore, *T*_1*ρ*_ has shown potential to probe diseases such as spinal disc degeneration,^33,34^ Alzheimer's disease,^35,36^ Parkinson's disease^37^ and ischemia.^38^

The conventional *T*_1*ρ*_ pulse sequence consists of a continuous wave (CW) radiofrequency (RF) pulse followed by the spin lock period and signal acquisition.^39–41^ Alternatively, the *T*_1*ρ*_ experiments can be performed using adiabatic spin locking.^42–44^ The conventional experiments must be repeated with incremented spin lock times, such that the total experiment time ranges from minutes to days depending on the number of repetitions. Furthermore, *T*_1*ρ*_ dispersion experiments are more time consuming since the experiments must be repeated with several SL frequencies. Anoardo *et al.* have presented an off-resonance technique (SLOAFI)^45^ for determining *T*_1*ρ*_ relaxation dispersion in a shorter time, but to our knowledge, no methods for acceleration of the *T*_1*ρ*_ preparation portion of the on-resonance rotating frame relaxation experiment have been reported. To circumvent the long experiment times, several rapid *T*_1*ρ*_ imaging methods have been developed. For example, Duvvuri *et al.*^46^ combined a fast spin echo sequence with *T*_1*ρ*_ preparation, Li *et al.*^47^ developed a *T*_1*ρ*_ imaging sequence based on spiral *k*-space readout, Liimatainen and Gröhn^48^ acquired *T*_1*ρ*_ decay of one *k*-space line during single adiabatic pulse train, and Bothakur *et al.*^49^ introduced a pulse sequence for spin locked echo planar imaging (SLEPI). There are also accelerated *T*_1*ρ*_ methods based on gradient echoes and steady state free precession,^50–53^ and reconstruction of undersampled *k*-space data.^54–60^ The development of rapid imaging methods is advantageous not only for reducing patient imaging time, but also for allowing imaging of dynamic objects like a cardiovascular system.^54–57^ In addition, the short experiment time of SLEPI has expanded the application of *T*_1*ρ*_ to functional MRI.^61^

Here, we introduce a novel single scan method for rotating frame relaxometry. In this method, the *T*_1*ρ*_ is produced by the SPIn lock CYcle (SPICY), in which a single RF excitation pulse is followed by a loop of spin locking and signal acquisition lobes. This allows for the determination of *T*_1*ρ*_ within a single scan, similar to the time domain Carr–Purcell–Meiboom–Gill (CPMG)^8^ acquisition, which measures *T*_2_. The total scan time is from a few seconds to minutes. We perform ^1^H *T*_1*ρ*_ dispersion experiments with both the conventional and the SPICY *T*_1*ρ*_ sequences on liquid samples and protein hydrogels prepared from the main constituents of the articular cartilage extracellular matrix, and we show that the relaxation time information obtained with these methods is in good agreement. In addition, we show that the SPICY sequence can be combined with spatial encoding to produce 1D images in a single scan.

## 2. Experimental

### 2.1. Samples

Three liquid samples, A, B and C, were used. The samples were made by dissolving CuSO_4_·5H_2_O in water (10% H_2_O, 90% D_2_O), with the sample A containing 0.013 wt% and sample B containing 0.04 wt% CuSO_4_. The solutions were placed in 5 mm NMR tubes. Sample C represents a two-compartment sample of liquids A and B made by placing the 0.04 wt% solution to the bottom of a 5 mm tube inside which a 3 mm tube containing the 0.13 wt% solution was placed. The tubes were placed in the RF coil in such a way that the liquid B covered the bottom half and liquids A and B the top half of the coil. The illustration of the samples is shown in Fig. 3f.

A hydrogel sample containing 40 mg g^−1^ collagen type II, 10 mg g^−1^ chondroitin sulfate and phosphate-buffered saline (PBS) was used. 10 mL of collagen solution (rat tail, ∼6 mg mL^−1^ in 0.01 M acetic acid, Merck KGaA, Darmstadt, Germany) was added to a sample tube together with 0.15 mL 10× PBS. Stock solution of CS (50–80 mg mL^−1^ CS powder from bovine trachea, Merck KGaA, Darmstadt, Germany in double distilled water) was added to the collagen solution and the mixture was diluted with double distilled water to give a total volume of 6 mL. The sample was thoroughly mixed and pH adjusted to basic (pH: 8–9) using 1 M sodium hydroxide (NaOH) and 0.5 M hydrochloric acid (HCl). The sample tube was placed for 45–60 min in a water bath at 37 °C to solidify the mixture into a gel. The solid was removed from the sample tube, transferred into a plastic cell culture dish, and dried at 40 °C in a laminar flow oven until the mass of the sample was reduced to 1.50 ± 0.03 g. Finally, the sample was placed in a 5 mm NMR tube in the middle of the RF coil.

### 2.2. Pulse sequence design

**Fig. 1:**
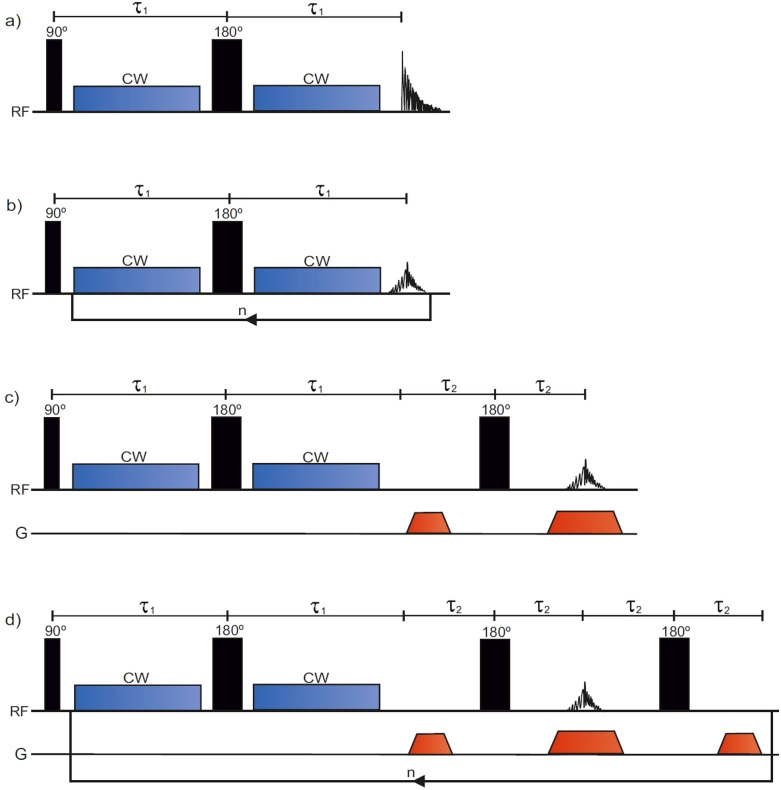
The pulse sequences for (a) conventional *T*_1*ρ*_, (b) SPICY, (c) conventional 1D *T*_1*ρ*_ imaging and (d) SPICY 1D imaging.

The conventional *T*_1*ρ*_ pulse sequence^39^ (Fig. 1a) consists of a 90° excitation pulse, two CW spin lock pulses separated by a 180° pulse, and an acquisition period in which the free induction decay (FID) is recorded. To encode for *T*_1*ρ*_, the sequence is repeated with incremented SL pulse durations. Contrary to the conventional *T*_1*ρ*_ pulse sequence, the SPICY pulse sequence (Fig. 1b) utilizes a loop structure which repeats the spin locking of constant length and signal acquisition blocks *n* times during one scan. To accomplish this, the acquisition period acquires the signal intensity of the spin echo. While in the conventional experiment *T*_1*ρ*_ is determined from the FID signal, in the SPICY, the *T*_1*ρ*_ fit is obtained from the echo intensities corresponding to different echo numbers. The 1D imaging versions of the conventional *T*_1*ρ*_ and the SPICY sequences are shown in Fig. 1c and d. In the SPICY imaging sequence, the third 180° pulse and the dephasing gradient block are needed to return to the center of the *k*-space before the beginning of the next loop. An 8-step phase cycle is used in the SPICY sequences (see the ESI†).

### 2.3. Experiments

The *T*_1*ρ*_ experiments were performed on a spectrometer with 600 MHz ^1^H operating frequency equipped with a 5 mm QXI probe with a gradient strength of 0.6 T m^−1^ and TopSpin 3.6.2 software (Avance III, Bruker Biospin, MA, USA). The probe temperature was set to 290 K during the experiments. In all the experiments, the 90° excitation pulse had a length of *P*_1_ ≈ 9.5 μs, and the 180° pulse had *P*_2_ ≈ 19 μs. The relaxation delay in all the experiments was 15 s. In all *T*_1*ρ*_ experiments, the SL-pulses were applied on-resonance. In the conventional *T*_1*ρ*_ experiments (Fig. 1a and c), the SL-pulse length was incremented between 30 and 480 ms in 16 steps. In the non-imaging SPICY measurements (Fig. 1b) of the water samples, the SL pulse duration was 30 ms and the number of echoes *n* was 16. The signal at the center of the echo was acquired for 50 μs with 12 points and the total SL interruption time during a single loop including all the SL gaps was 130 μs. In the SPICY measurements of hydrogels, the SL pulse duration was 40 ms, the number of echoes was 12, the signal acquisition time was 25 μs, the number of points was 6 and the total SL interruption time was 100 μs. In the SPICY imaging sequence (Fig. 1d), the signal at the center of the echo was acquired for 0.82 ms with 512 points and the total SL interruption time was 8 ms. In both imaging sequences, the read gradient length was 0.82 ms, the gradient ramp time was 100 μs, and the gradient strength was 0.3 T m^−1^. The length of the dephasing gradients was half of the read gradients. The non-imaging sequences were performed with SL frequencies of 2–11 kHz and the imaging experiments with 7 kHz. The number of scans was 16 for the water samples and 32 for the hydrogel sample. The experiment time of one scan was 4 min 12 s for the conventional and 16 s for the SPICY sequences.

### 2.4. Data analysis

To find *T*_1*ρ*_ relaxation times, the equation 
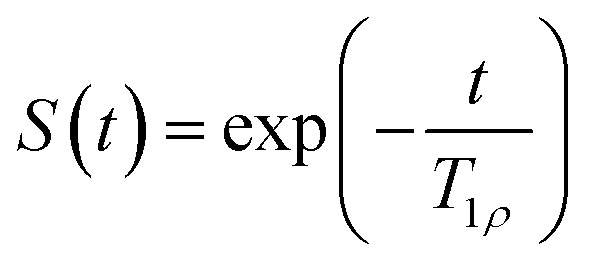
 was fitted to the experimentally obtained signal decay. In the analysis of the SPICY data, the signal was summed across all the points acquired for each echo to perform *T*_1*ρ*_ fitting while the area under the spectra was used in the conventional *T*_1*ρ*_ analysis. The 1D images were obtained by Fourier transform. The data analysis was done using MATLAB (Mathworks, R2020b, Natick, MA, USA).

## 3. Results and discussion

**Fig. 2:**
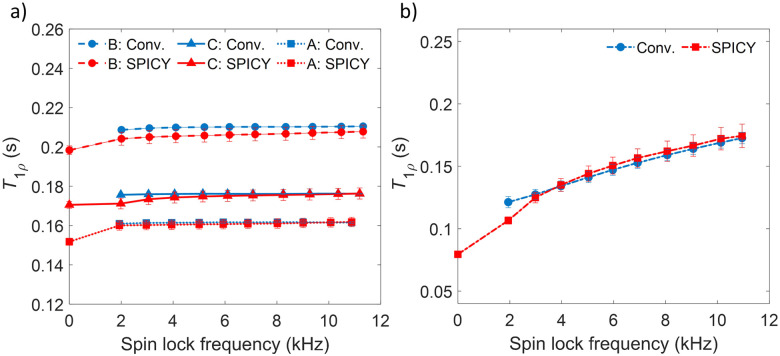
(a) *T*_1*ρ*_ measured with different spin lock frequencies for the water samples A, B and C. (b) *T*_1*ρ*_ dispersion measured for the hydrogel sample. Blue color indicates the conventional *T*_1*ρ*_ and red SPICY. The *T*_1*ρ*_ value measured with 0 kHz SL frequency is the *T*_2_ of the sample.

To determine the accuracy of the single scan acquisition method, the *T*_1*ρ*_ relaxation times recorded using the SPICY method were compared to the *T*_1*ρ*_ relaxation times recorded using the two-dimensional conventional acquisition scheme (Fig. 2). The *T*_1*ρ*_ values were measured for three aqueous solutions A, B and C with variable SL fields in the range of 2–11 kHz (Fig. 2a). The *T*_2_ values were measured for all samples with SPICY sequence by turning off the CW pulses. All samples show stable *T*_1*ρ*_ values over the whole frequency range, and the difference between conventional and SPICY *T*_1*ρ*_ values is less than 2% for all three aqueous solutions, which indicates that the SPICY sequence is precise and works under different conditions.

**Fig. 3:**
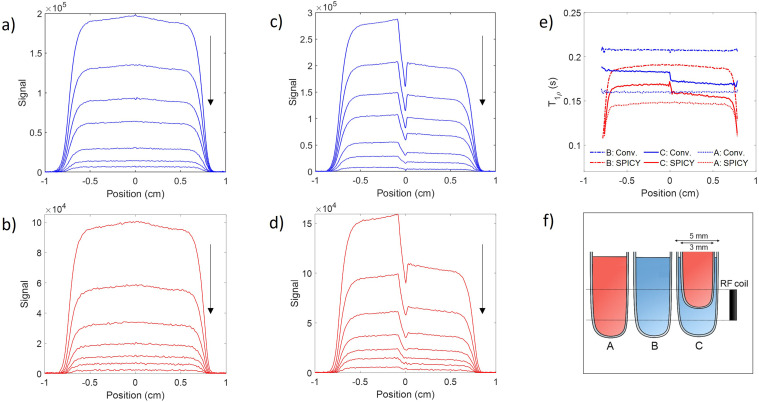
(a–d) 1D MR images of the samples A (left; a and b) and C (right; c and d) measured with the conventional (blue; a and c) and the SPICY *T*_1*ρ*_ (red; b and d) sequences. The arrow indicates the direction of increasing spin lock time (a and c) or echo number (b and d). (e) *T*_1*ρ*_ profiles of samples A (dotted line), B (dashed-dotted line) and C (solid line) measured with the conventional (blue) and the SPICY sequence (red). (f) Illustration of samples A, B and C with respect to the RF coil.

To test the applicability of the SPICY sequence on a biochemically more relevant sample, *T*_1*ρ*_ dispersion was measured for the hydrogel sample containing collagen and chondroitin sulfate which acts as a model of the cartilage extracellular matrix (Fig. 2b). The SL frequency was varied between 2–12 kHz leading the *T*_1*ρ*_ values to increase between 0.12–0.17 s. The difference between the SPICY and reference *T*_1*ρ*_ values is only 1–2% showing that the SPICY method is applicable to *T*_1*ρ*_ dispersion studies of more complicated samples as well. The SPICY sequence was further tested with imaging such that 1D read gradients were used to record 1D signal intensity data (Fig. 1d and 3). The 1D images of the samples A and C measured with the conventional and the SPICY readouts are shown in Fig. 3a–d. The image profiles obtained with both methods are consistent. The *T*_1*ρ*_ profiles measured for all three samples are shown in Fig. 3e. The *T*_1*ρ*_ values obtained with SPICY imaging are about 8% smaller than the values measured with the conventional imaging sequence. The small difference is expected to arise predominantly from the signal decay caused by molecular diffusion in the presence of gradients, because the SPICY imaging experiment includes more gradient pulses due to the additional rephasing gradient and loop structure.^62^ The difference can be minimized by minimizing the gradient strength and length. Both methods give stable profiles, however the *T*_1*ρ*_ values measured with SPICY show decreased *T*_1*ρ*_ values at the edges of the coil. This is a consequence of a decrease of the *B*_1_ field close to the edges of the coil and much higher number of 180° pulses in the SL gaps during the SPICY experiment. One of the major differences between the principles of SPICY and conventional sequences is the *T*_2_ decay occurring during the SL gaps during the SPICY experiment. For that reason, the delays between the SL pulses and the signal acquisition period should be set as short as possible. Especially samples with strong dispersion can be very sensitive to non-locked periods, which is why *T*_2_ relaxation should be considered when performing the SPICY measurements. In the SPICY imaging sequence, which includes the longer acquisition period due to the imaging gradients, the *T*_2_ decay affects the signal more than in the non-imaging experiments. The *T*_2_ effect can be reduced either by shortening the non-locked periods or by reducing the number of echoes and increasing the SL pulse length. Since the acquisition time of the echo signal should be short, the SPICY sequence cannot be used to resolve spectral lines by Fourier transform.

The SPICY method has a lot of potential for future applications in both spectroscopy and imaging. The SPICY method could be applied to accelerate the *T*_1*ρ*_ relaxation dispersion studies of biomacromolecules and to determine, for example, the rates of chemical exchange or conformational changes.^4–7,11–22^ We showed dispersion measurements with SL fields of 2–11 kHz, however, a wider range of SL fields could be applicable. The SPICY sequence could be further developed for accelerating multidimensional *T*_1*ρ*_ imaging by adding additional spatial encoding dimensions, for example, in the same way as in the RARE imaging.^63^ Because SPICY is much faster and the spin locking does not produce more energy deposition compared to the conventional method, it could have a lot of potential in clinical MRI. The higher number of 180° pulses may cause more heating compared to the standard method, however, the problem could be avoided by replacing the image readout part by gradient echo. The *T*_1*ρ*_ values of the SPICY imaging remain slightly smaller than the reference values due to the higher number of gradient pulses and longer SL gap during signal acquisition, but it must be noted that in clinical imaging, the contrast between tissues is generally more important than the quantitative *T*_1*ρ*_ values. Also, the decreased *T*_1*ρ*_ values at the edges of the RF coil can be managed by placing the imaged object in the middle of the sensitive area of the coil. In the same way as the SLEPI sequence,^49,60^ SPICY could be applied to imaging of dynamic processes. It might be possible to use adiabatic spin locking in the SPICY sequence.^42–44^ Because the SPICY allows signal readout in a similar manner to the CPMG,^8^ it could be added to multidimensional NMR or Laplace NMR experiments^64^ to correlate *T*_1*ρ*_ with the other relaxation or diffusion parameters. Furthermore, the 1D imaging version of SPICY could replace the CPMG readout part in multidimensional ultrafast NMR^65^ or Laplace NMR (LNMR) experiments.^62,66–69^ The single scan approach also significantly facilitates the use of nuclear spin hyperpolarization techniques^70–73^ to boost the sensitivity of the experiments by several orders of magnitude. For example, recently Qi *et al.*^74^ boosted *T*_2_ relaxation dispersion measurements by dissolution dynamic nuclear polarization (dDNP) to study protein–ligand binding. Similarly, SPICY could be exploited in hyperpolarized studies as well.

## 4. Conclusions

In summary, we introduced a novel single-scan method called SPICY for measuring *T*_1*ρ*_ relaxation times. We demonstrated that the SPICY method can be successfully used to measure *T*_1*ρ*_ dispersions with spin locks of 2–11 kHz. SPICY shortens the scan time about an order of magnitude while producing less RF energy deposition compared to the conventional *T*_1*ρ*_ sequence. We showed that the *T*_1*ρ*_ values obtained from the SPICY sequence are in good agreement with those of the conventional method. We also combined the SPICY with one dimensional spatial encoding and showed that the method is applicable to single scan 1D imaging as well. Overall, the single scan *T*_1*ρ*_ method presented in this work has a lot of potential for future applications in rotating frame relaxation studies, MR imaging, ultrafast multidimensional NMR and Laplace NMR studies, as well as in hyperpolarize NMR and MRI experiments.

## Author contributions

K. Tolkkinen was responsible for conceptualization, methodology, validation, investigation, resources, and writing – original draft. S. Mailhiot was responsible for conceptualization, methodology, validation, investigation, and writing – review and editing. A. Selent was responsible for conceptualization, methodology, and writing – review and editing. O. Mankinen was responsible for support in the final measurements, and writing – review and editing. H. Henschel, M. Nieminen and M. Hanni were responsible for planning and preparing the hydrogel sample, and writing – review and editing. A. M. Kantola was responsible for supervision, and writing – review and editing. T. Liimatainen was responsible for supervision, and writing – review and editing. V.-V. Telkki was responsible for conceptualization, supervision, and writing – review supervision, and writing – review and editing.

## Conflicts of interest

There are no conflicts to declare.

## Supplementary Material

CP-025-D2CP05988F-s001
